# Clinical Analysis of C-Shaped Embedded Pancreaticojejunostomy in Pancreaticoduodenectomy

**DOI:** 10.1155/2022/7427146

**Published:** 2022-05-27

**Authors:** Hui Huang, Jichun Sun, Zhiqiang Li, Longjun Zang, Hongwei Zhu, Xianlin Zhang, Xiao Yu

**Affiliations:** ^1^Department of Hepatopancreatobiliary Surgery, The Third Xiangya Hospital, Central South University, Changsha, 410013 Hunan, China; ^2^Department of General Surgery, Affiliated Renhe Hospital of China Three Gorges University, Yichang, 443001 Hubei, China

## Abstract

**Background:**

Comparing the effects of C-shaped embedded anastomosis and pancreatic duct-jejunal mucosal anastomosis on the incidence of pancreatic fistula after pancreaticoduodenectomy (PD) to find a better pancreaticojejunal anastomosis method that can reduce the occurrence of complications during the operation and benefit the patients.

**Methods:**

A retrospective subresearch method was used to select the clinical data of patients who have undergone pancreaticoduodenectomy in our hospital from December 2019 to March 2021. The indicators to be collected for this study include gender, age, body mass index, preoperative liver function (total bilirubin, alanine aminotransferase, and albumin), preoperative comorbidities (diabetes, chronic pancreatitis), and pancreatic condition (texture, pancreatic duct diameter). The patients were divided into two groups according to the method of pancreaticojejunostomy: C-shaped embedded anastomosis group (*n* = 38) and pancreatic duct-jejunal mucosal anastomosis group (*n* = 30). The duration of pancreaticojejunostomy, biliary-enteric anastomosis, gastrointestinal anastomosis, intraoperative blood loss, upper abdominal surgery history, pathological type, intraoperative blood loss, pancreaticojejunostomy time, combined pancreatic fistula, biliary fistula, hemorrhage, and abdominal infection were observed and compared. According to the different methods of pancreaticojejunostomy during operation, they were divided into group A: C-shaped embedded pancreaticojejunostomy group (38 cases), and group B: pancreatic duct-jejunal mucosal anastomosis group (30 cases). The postoperative complications were compared between the two groups, and the observed indicators were analyzed with statistical methods.

**Results:**

The average pancreaticojejunostomy time in group A was 32.13 ± 4.52 min, and the average pancreaticojejunostomy time in group B was 43.23 + 4.31 min. The difference was statistically significant (*p* < 0.05). Neither group A nor group B had a grade C fistula. The incidence of biochemical fistula in group A was 21.05% (8/38), and the incidence of biochemical fistula in group B was 13.3% (4/30). The difference was not statistically significant (*p* > 0.05). The incidence of grade B fistula in group A was 5.20% (2/38), and the incidence of grade B fistula in group B was 26.67% (8/30). The difference was statistically significant (*p* < 0.05). There were no perioperative deaths in the two groups.

**Conclusion:**

According to the results of data analysis, it can be seen that both the two types of pancreaticojejunostomy have good clinical effects, but that in terms of reducing the grade of pancreatic fistula, the C-shaped embedded pancreaticojejunostomy is obviously better and safer. At the same time, the C-shaped embedded pancreaticojejunostomy can shorten the time of pancreaticojejunostomy and is easier to operate, thus worthy of clinical promotion.

## 1. Introduction

Pancreatic ductal adenocarcinoma (PDAC) is one of the most deadly tumors in gastrointestinal tumors and is also one of the four most common causes of cancer-related deaths [1]. Although the current molecular biology, medical system, treatment technology, and chemotherapy have made great progress, the 5-year survival rate of pancreatic cancer is only 12%. At present, surgical RO resection is still the only possible cure for pancreatic cancer [2, 3]. Pancreaticoduodenectomy, also known as Whipple Operation, is the current standard procedure for the treatment of malignant tumors of the pancreatic head and ampulla. In 1898, Codivilla, an Italian physician, for the first time performed pancreaticoduodenectomy (PD), which has been the standard surgical operation for the treatment of benign and malignant lesions in the areas around pancreatic head and ampulla. The reconstruction of digestive tract is the key in the operation, and plays a decisive role in the recovery of patients. With the rapid development of surgical techniques and perioperative management concepts in the past 20 years, the operative mortality rate of pancreaticoduodenectomy has dropped to less than 5% [4]. But the incidence of postoperative complications is still high, because the surgical procedure is complicated, involving quite many organs, and the reconstruction steps are intricate [5]. According to reports from dozens of medical centers in different countries, the incidence of postoperative complications after pancreaticoduodenectomy is 30%-60% [6]. Common postoperative complications include postoperative pancreatic fistula (PF), delayed gastric emptying (DGE), and postpancreatectomy hemorrhage (PPH) [7]. Among them, pancreatic fistula is most common, which can cause a significant increase in hospital stay and hospitalization costs, can further lead to severe consequences such as postoperative abdominal bleeding and infection, and can even endanger the patients' lives [8]. Therefore, in the perioperative management, the prevention and treatment of pancreatic fistula are of high priority [9]. In order to better prevent and reduce the incidence of postoperative pancreatic fistula, pancreatic surgeons have invented a variety of approaches for pancreaticojejunostomy. However, the best one has not been found yet. Surgeons have untiringly tired various possible effective methods to reduce the occurrence of PF, such as insertion of drainage tube in the pancreatic duct, preoperative reduction of jaundice, and application of somatostatin [10–13]. However, the general conditions of patients before surgery, such as gender, age, and whether with diabetes or chronic pancreatitis, are objective and unchangeable. Therefore, the treatment of the pancreatic stump as a controllable factor seems to be particularly important in reducing the occurrence of PF. Theoretically, pancreaticogastrostomy (PG) has more advantages, but most studies currently show that the incidence of PF after PG is not lower than that of pancreaticojejunostomy (PJ), so most surgeons still favor the PJ method, which mainly includes invagination pancreaticojejunostomy (IPJ) and duct-to-mucosa pancreaticojejunostomy (DmPJ) [14–16]. Based on these, many scholars have improved and innovated a large number of PJ methods, with more than 100 methods currently. The author's team has improved and innovated on this basis and gradually formed its own PJ method, which is “C-shaped embedded pancreaticojejunostomy.” The main purpose of this article is to compare the occurrence of PF and other complications of the two anastomosis methods, C-shaped embedded pancreaticojejunostomy and pancreatic duct-jejunal mucosal anastomosis, so as to provide reference for future clinical practice.

## 2. Data Collection

### 2.1. Inclusion Standard

The following are the inclusion standards: (1) The subjects have been informed and agreed. (2) The subjects have a complete preoperative laboratory examination, and the location of the lesion requires pancreaticoduodenectomy. (3) The patients are over 18 years old and have a clear consciousness and language expression. (4) All indicators of renal and kidney functions shall meet surgical standards within one week of surgery

### 2.2. Exclusion Standard

The following are the exclusion standards: (1) Metastasis has occurred in distant organs (liver, lung, and abdominal cavity), surrounding tissues, and lymph nodes; the tumor has adhered to many surrounding tissues. (2) Patients have complicated severe dysfunction in organs such as the heart, lung, liver, and kidney. (3) Patients have human immunodeficiency virus (HIV). (4) The pancreaticojejunostomy adopts an anastomosis method other than the two discussed in this study. (5) Patients have a history of major surgery or the resulting complications having not been cured 3 weeks before surgery. (6) Patients have incomplete clinical data

### 2.3. Case Data

The clinical data of 68 patients having undergone pancreaticoduodenal anastomosis in our hospital from December 2019 to March 2021 were collected. Among them, 35 are males and 33 are females. The approximate age range of the patients is 45-77 years, and the average age is 61.2 ± 8.93 years. Postoperative pathology showed the following: 10 cases with pancreatic head cancer, 14 cases with duodenal ampullary carcinoma, 10 cases with pancreatic ductal adenocarcinoma, 23 cases with distal bile duct cancer, and 11 cases with pancreatic head mass pancreatitis.

### 2.4. Grouping

According to the different anastomosis methods, they are divided into group A: C-shaped embedded anastomosis, and group B: pancreatic duct-jejunal mucosal anastomosis.

## 3. Preoperative Preparation

The following is the preoperative preparation:

1. Rehydration is performed to correct acid-base imbalance and electrolyte ion disorder
2. Improving preoperative anemia, hypoproteinemia, and jaundice and for patients unable to eat, intravenous nutritional support shall be given
3. Liver protection treatment was performed for patients with abnormal liver function, and the patients shall be advised to eat more high-protein foods before surgery
4. For patients complicated with basic diseases such as heart disease, hypertension, and diabetes, relevant departments shall be invited for consultation, and the patient's diet will be adjusted and arranged based on the consultation's opinions to ensure the patient can undergo surgery normally
5. Before operation, food and water are prohibited, a gastric tube will be placed, and broad-spectrum antibiotics will be applied 0.5-1 hour before operation to prevent infection

## 4. Surgical Approach

### 4.1. C-Shaped Embedded Anastomosis Method

**Figure 1:**
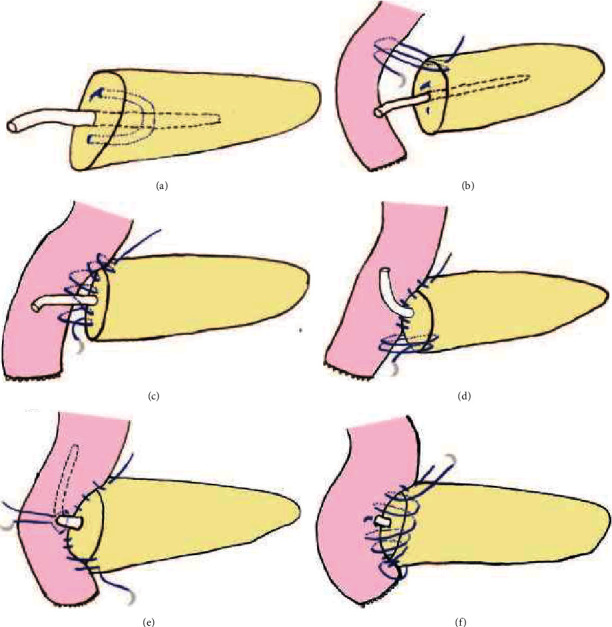
Illustrations of C-shaped embedded anastomosis. (a) Perform U-shaped suture to fix the drainage tube in the pancreatic duct. (b) Suture the whole upper pancreas edge with the seromuscular layer of the jejunum twice. (c) Suture the posterior wall of the pancreas and the seromuscular layer of the jejunum with a continuous suture. (d) Suture the whole lower pancreas edge with the seromuscular layer of the jejunum twice. (e) Perform a purse string suture around the incision of the jejunum. (f) Suture the anterior wall of the pancreas and the jejunum.

**Figure 2:**
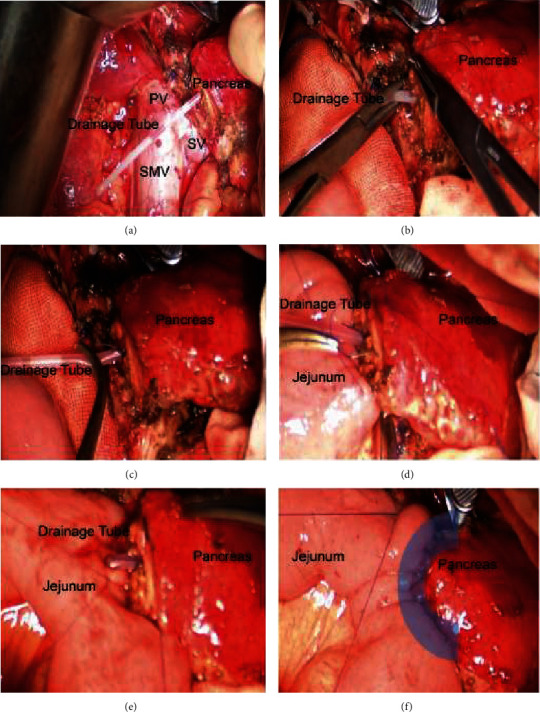
Surgical photos of C-shaped embedded anastomosis. (a) Insert the pancreatic duct drainage tube into the main pancreatic duct for about 6-7 cm. (b) U-shaped suture the drainage tube in the pancreatic duct. (c) Make sure the drainage tube is fixed. (d) Suture whole lower pancreas edge with the seromuscular layer of the jejunum. (e) The jejunum wrapped the upper, posterior, and lower edges of the pancreas by about 1 cm. (f) Suture the anterior wall of the pancreas and the jejunum to form a C-shaped embedded anastomosis. PV: portal vein; SMV: super mesenteric vein; SV: splenic vein.

A C-shaped embedded anastomosis method was used in group A. Insert the pancreatic duct drainage tube into the main pancreatic duct for about 6-7 cm, and use a 15 cm 3.0 absorbable thread to penetrate the pancreas, pancreatic duct, and pancreatic duct drainage twice to form a U-shaped suture which fixed the tube in the pancreatic duct (see Figure 1(a)). Use a 50 cm 3.0 Prolene thread to penetrate the whole pancreas at pancreas superior margin and anastomosis with the seromuscular layer of the jejunum twice (see Figure 1(b)). Go on to suture the posterior wall of the pancreas and the seromuscular layer of the jejunum with a continuous suture (see Figure 1(c)); then penetrate the whole pancreas at pancreas inferior margin and anastomosis with the seromuscular layer of the jejunum twice (see Figure 1(d)) so that the jejunum wrapped the upper, posterior, and lower edges of the pancreas by about 1 cm to form a C-shaped embedded anastomosis. Use an electric hook to make a 0.5 cm incision at the edge of the mesangium of the jejunum, and then insert the pancreatic duct drainage tube into the cavity of the jejunum for about 10 cm through the incision. Then, use a 15 cm 3.0 absorbable thread to perform a purse string suture around the incision of the jejunum (see Figure 1(e)). After tightening the knot, continue with the first 3.0 Prolene thread to suture the anterior wall of the pancreas and the jejunum to complete the C-shaped embedded anastomosis of the pancreas and jejunum (see Figure 1(f)). Finally, we also captured the key steps of the operation to supplement the description of the anastomosis method, as shown in Figure 2.

### 4.2. Pancreatic Duct-Jejunal Mucosal Anastomosis

Pancreatic duct-jejunal mucosal anastomosis method was used in group B. First, after marking the pancreatic duct of the patients in the conventional group, suture the dorsal side of the pancreatic stump and the seromuscular layer corresponding to the jejunum with a 3.0 Prolene thread about 2 cm from the margin of the pancreas; make a small hole in the jejunum wall near the main pancreatic duct; expand the diameter of the small hole consistent with the diameter of the pancreatic duct. Use a 5.0 Prolene thread to suture the mucosa of the posterior wall of the small hole and the posterior wall of the pancreatic duct with interrupted sutures; choose a suitable silicone support tube to stay in the pancreatic duct, insert the other end into the hole of the intestinal wall of the jejunum, and use a 5.0 Prolene thread to suture the anterior wall mucosa of the jejunal orifice and the anterior pancreatic duct mucosa with interrupted sutures; use a 3.0 Prolene thread to suture the dorsal membrane of the pancreatic stump and the seromuscular layer of the anterior wall of the jejunum with interrupted sutures.

### 4.3. Observation Index

The following are the observation indexes: (1) perioperative indicators; (2) postoperative complications: the incidence of different grades of pancreatic fistula and the incidence of delayed gastric emptying, bile leakage, abdominal hemorrhage, and abdominal infection (as for the pancreatic fistula grading and evaluation standard, refer to the diagnostic standard issued by the International Study Group of Pancreatic fistula (ISGPF) in 2016 [3]); and (3) the concentration of amylase in the abdominal drainage fluid after surgery

### 4.4. Diagnostic Standard for Complications

The diagnostic standard of the study was all based on the “Expert Consensus on the Diagnosis, Treatment and Prevention of Common Diseases after Pancreatic Surgery (2017).”

#### 4.4.1. Diagnosis of Postoperative Pancreatic Fistula

Any measurable content of amylase in the abdominal drainage fluid exceeding 3 times the upper limit of normal serum amylase, and accompanied by clinical symptoms related to PF, can be diagnosed as pancreatic fistula. According to different standards, it is divided into biochemical fistula, grade B fistula, and grade C fistula (see Table 1).

#### 4.4.2. Diagnosis of Postoperative Biliary Fistula

According to ISGPS definition, biliary fistula can be diagnosed if the concentration of bilirubin in abdominal drainage fluid exceeds 3 times of the upper limit of normal blood bilirubin concentration at any time from the third day after surgery. Imaging examination suggested that fluid accumulation around the biliary anastomosis could provide indirect evidence for biliary fistula and provide guidance for the treatment of biliary fistula. Patients with biliary fistula often have higher amylase levels due to the presence of pancreatic fluid in the intestinal cavity (see Table 2).

#### 4.4.3. Postoperative Delayed Gastric Emptying

In this study, the diagnostic standard for postoperative gastric emptying delay adopted the postpancreatectomy delayed gastric emptying (DEG) standard proposed by the International Study Group on Pancreatic Surgery (ISGPs). Before diagnosis, the abnormal gastric emptying caused by mechanical obstruction should be ruled out, and the gastrojejunal or duodenal-jejunal anastomosis should be unobstructed by endoscopy or upper gastrointestinal angiography (see Table 3).

#### 4.4.4. Diagnosis of Postoperative Hemorrhage

In this study, the postoperative hemorrhage classification adopted the postpancreatectomy hemorrhage standard proposed by the International Study Group on Pancreatic Surgery (ISGP). According to the bleeding time, it is divided into early postoperative hemorrhage (less than or equal to 24 h after surgery) and late postoperative hemorrhage (more than 24 h after surgery) (see Table 4).

- Grade A: there is no change in the examination results and postoperative conditions, and the patient can be discharged from hospital at scheduled time.
- Grade B: there are changes in the examination results and postoperative conditions, and these affect postoperative management, which may require blood transfusion, ICU treatment, and invasive operations.
- Grade C: it severely affects the clinical results, even life-threatening, and affects postoperative management, which requires to prolong postoperative hospital stay and ICU time.

#### 4.4.5. Diagnosis of Postoperative Abdominal Infection

It was evaluated according to the expert Consensus on the Prevention and Treatment of Common postoperative complications of pancreas proposed by the Pancreatic Surgery Group of Chinese Surgical Society [17]. The diagnostic criteria of postoperative intraperitoneal infection were as follows: (1) fever (*T* > 38.0°C), abdominal pain, abdominal distension and obvious signs of peritonitis appeared three days after surgery, and the white blood cell count which was >10 × 10^9^/L. (2) The abdominal drainage fluid was purulent and positive in bacteriological culture. (3) Imaging examination or reoperation confirmed the presence of infectious lesions in the abdominal cavity, such as suppurative exudation and abscess (see Table 5).

### 4.5. Statistical Methods

The statistical software SPSS26.0 was used for data analysis. All measurement data conforming to the normal distribution were described and represented by the mean ± standard deviation (*χ* ± *s*), and the difference between the groups was performed by two independent sample *t* tests; measurement data such as postoperative complications were performed by a *χ*^2^ test. The difference was statistically significant with *p* < 0.05.

## 5. Results

### 5.1. Comparison of Preoperative Situation of Patients with C-Shaped Embedded Anastomosis and Pancreatic Duct-Jejunal Mucosal Anastomosis

68 patients were included in this study, of which 38 patients underwent C-shaped embedded anastomosis and 30 patients underwent pancreatic duct-jejunal mucosal anastomosis. The average age of all patients was 61.98 ± 7.98 years old. As shown in the table, the preoperative albumin level of patients with C-shaped embedded anastomosis (hereinafter referred to as group A) was 34.89 ± 2.54 g/L, and the preoperative albumin level of patients with pancreatic duct-jejunal mucosal anastomosis (hereinafter referred to as group B) was 34.71 ± 4.08 g/L. The two groups had no statistical significance after statistical analysis. The preoperative serum bilirubin level of group A was 116.89 ± 59.97 *μ*mol/L, and the preoperative serum bilirubin level of group B was 97.64 ± 30.11 *μ*mol/L. The two groups had no statistical significance after statistical analysis. The average intraoperative blood loss in group A was 432.61 ± 205.38 mL, and the average intraoperative blood loss of group B was 481.38 ± 142.67 mL. The two groups had no statistical significance after statistical analysis (*p* = 0.078). Generally speaking, the two groups had no statistical significance in terms of preoperative general conditions, pancreatic conditions (texture, pancreatic duct diameter), intraoperative blood loss, and case types (*p* > 0.05). The specific results are shown in Table 6.

### 5.2. Comparison of Intraoperative Situation of Patients with C-Shaped Embedded Anastomosis and Pancreatic Duct-Jejunal Mucosal Anastomosis

In this study, the average operation time (anesthesia recording time) of group A was 4.67 ± 0.91 hours, and the average operation time of group B was 5.64 ± 1.03 hours. After statistical analysis, there was a statistical difference between the two groups (*p* = 0.041). The average number of lymph nodes removed in group A was 19.87 ± 2.32 during operation, and the average number of lymph nodes removed in group B was 17.85 ± 6.18 during operation. After statistical analysis, there was no statistical difference between the two groups (*p* = 0.235). The specific results are shown in Table 7.

### 5.3. Comparison of Postoperative Situation of Patients with C-Shaped Embedded Anastomosis and Pancreatic Duct-Jejunal Mucosal Anastomosis

Statistical analysis showed that among the 38 patients who have undergone C-shaped embedded anastomosis, there were 8 cases of biochemical fistulas (21.05%) and 2 cases of grade B fistulas (5.20%) in group A, and there were 3 cases of biochemical fistula (10%) and 8 cases of grade B fistula (26.66%) in group B. The difference between the two groups was not statistically significant in the comparison of biochemical fistula, while the difference was statistically significant (*p* < 0.05) in the comparison of grade B fistula. And there was no significant difference in the incidence of bile leakage, abdominal bleeding, delayed gastric emptying, and abdominal infection between group A and group B. The specific results are shown in Table 8.

## 6. Discussion

At present, cancer has become one of the main factors affecting public health. Pancreatic cancer is the most common one with high degree of malignancy. Since the early diagnosis rate is not high, its mortality rate ranks the fourth in all cancers globally. The National Comprehensive Cancer Network (NCCN) recommends pancreaticoduodenectomy (PD) as the only potential cure for pancreatic cancer in the clinical practice guidelines. In 1994, Dr. Ganger and Dr. Pomp from Canada performed the world's first laparoscopic pancreaticoduodenectomy (LPD) for a patient with chronic pancreatitis. Since then, more and more surgeons have begun to pay attention to the therapeutic effect of pancreaticoduodenectomy and innovate its anastomosis methods. However, there is still certain controversy regarding the safety, operability, and superiority of pancreaticoduodenectomy.

Pancreaticoduodenectomy is a relatively complicated surgical method, which can greatly affect the body and potentially cause a lot of complications. In recent years, more and more innovative anastomosis methods have been proposed, the safety of the perioperative period has been greatly improved, and the techniques have become more mature. It has become a commonly used surgical method in high-volume centers. Pancreaticojejunostomy is the most commonly used gastrointestinal reconstruction method in pancreaticoduodenectomy, and it is also a key factor determining the effect of surgery. Over the years, pancreatic surgeons have made multiple attempts in the methods and techniques of pancreaticojejunostomy. Various pancreaticojejunostomies emerged one after another, all aimed at achieving biological healing through a firm mechanical anastomosis. However, there is no pancreaticojejunostomy that can completely avoid pancreatic fistula so far. Among them, the pancreatic duct-to-jejunal mucosal anastomosis is achieved by suturing the pancreatic duct mucosa and the jejunum mucosa and making the pancreas section and the jejunum serous membrane closely attached. Theoretically, it can effectively prevent the occurrence of pancreatic fistula and related complications and become the first choice for pancreatic anastomosis. However, the results of recent prospective studies and meta-analysis showed that the pancreatic duct-to-jejunal mucosal anastomosis has not significantly reduced the incidence of pancreatic fistula, and for patients with soft pancreas or small pancreatic duct diameter (<3 mm), it requires considerable techniques and experience to conduct pancreatic duct-to-jejunal mucosal anastomosis. If not, it is more likely to cause serious complications such as postoperative pancreatic fistula. In recent years, with the gradual development of laparoscopic and robotic pancreaticoduodenectomy, the pancreaticojejunostomy has encountered a technical bottleneck. The traditional open pancreaticojejunostomy method is not suitable for minimally invasive pancreaticojejunostomy. How to conduct a minimally invasive pancreaticojejunostomy easily and safely has become the exploration target of pancreatic surgeons. Excessively tight pancreaticojejunostomy in the past can affect the blood supply of the anastomosis, excessive anastomotic tension can cause pancreatic tissue edema, resulting in severe suture cutting injury, and multilayer and overdense sutures can also cause penetrating injury to the pancreas, resulting in pancreatic juice leakage through the needle hole to form a pancreatic fistula. Based on the above understanding of traditional pancreaticojejunostomy, combined with a large number of laparoscopic and robotic pancreaticojejunostomy experiences, the author has gradually formed a simple and convenient C-shaped embedded pancreaticojejunostomy through continuous optimization of the suture method.

The results of this study showed that the average time of pancreaticojejunostomy in patients who underwent C-shaped embedded pancreaticojejunostomy was 32.13 ± 4.52 min, while the average time of pancreaticojejunostomy was 43.23 ± 4.31 min in patients who underwent pancreatic duct-jejunal mucosal anastomosis. The difference was statistically significant (*p* < 0.05). Neither of the two groups of patients had a Grade C fistula, but it can be clearly found from the data that in terms of reducing the grade of pancreatic fistula, the C-shaped embedded pancreaticojejunostomy is better and safer. There was no perioperative death in the two groups. In terms of operation time, the C-shaped embedded pancreaticojejunostomy was also relatively shorter and easier to operate. There were few patients with gastric emptying disorders, bile leakage, and abdominal infections after surgery, and none of the remaining patients had complications required surgical intervention. In this study, due to limited conditions, not many patients were included. At the same time, the procedure is currently in the learning curve stage. However, from the previous results, this procedure is efficient in time and simple in steps, which is very suitable for large-scale clinical applications. It can not only significantly reduce the incidence of postoperative pancreatic fistula and related complications but is also a safe and effective method of pancreaticojejunostomy. If its role in reducing the incidence of postoperative pancreatic fistula can be further confirmed in a large sample of patients, it will be revolutionary in the change of the concept of pancreaticojejunostomy and the promotion of pancreaticoduodenectomy. The C-shaped embedded pancreaticojejunostomy is simple and convenient to operate and strives to achieve the fastest biological healing with minimal mechanical damage. The suture of the pancreas section and the jejunum serosa muscle layer can be achieved with one thread, which with an assistant U shaped suture and purse suture, still simplified the whole process a little longer and guarantee a reduced rate of postoperative complication.

## 7. Conclusions

The C-shaped embedded anastomosis method proposed in this study is an innovative anastomosis method based on the original pancreaticojejunal end-to-side anastomosis. By including the cases of both pancreatic duct-jejunum anastomosis and C-shaped embedded anastomosis methods, the data further confirmed that the C-shaped embedded anastomosis method is safe and feasible. Besides, it also has the advantages of less hemorrhage, shorter postoperative hospital stay, and lower hospitalization costs. Compared with pancreaticojejunal anastomosis, C-shaped embedded anastomosis has higher technical advantages for doctors and lower perioperative mortality and postoperative complications for the patient. All in all, the anastomosis method is simple and easy to operate, the incidence of postoperative pancreatic fistula is low, and it has good safety and effectiveness. It is suitable for promotion and application in both open and minimally invasive pancreaticoduodenectomy.

## Figures and Tables

**Table 1:** Classification of postoperative pancreatic fistula.

Events	Biochemical fistula	Grade B fistula	Grade C fistula
Drainage fluid amylase concentration	>3 times the upper limit of normal value	>3 times the upper limit of normal value	>3 times the upper limit of normal value
Peripancreatic drainage time	≤3 weeks	>3 weeks	>3 weeks
Postoperative management changes	No	Yes	Yes
Puncture/endoscopic interventional drainage	No	Yes	Yes
Bleeding/contrast embolization	No	Yes	Yes
PF-related infections	No	Yes, without organ failure	Yes, with organ failure
PF-related secondary surgery	No	No	Yes
PF-related organ failure	No	No	Yes
PF-related deaths	No	No	Yes

**Table 2:** Diagnosis of postoperative biliary fistula.

Diagnostic basis
Clinical manifestations: symptoms of infection, peritoneal irritation sign
Laboratory examination: water electrolytes acid-base imbalance, increased blood alkaline phosphatase, increased direct bilirubin, increased blood cell count
Imaging examination: localized effusion in the biliary-enteric anastomosis
Diagnosis: diagnosed abdominal puncture (puncture and extract bile under CT or ultrasound guidance)

**Table 3:** Data.

Grading	Indwelling gastric tube for decompression	Eat solid food	Abdominal distention or vomiting	Prokinetic drugs
Grade A	4-7d or reinsertion	<13d	Yes/no	Yes/no
Grade B	8-14d or reinsertion	14-21d	Yes	Yes
Grade C	>14d or reinsertion	>21d	Yes	Yes

**Table 4:** Diagnosis of postoperative hemorrhage.

Diagnostic basis
Clinical manifestations: increased heart rate, increased blood pressure, decreased urine output, increased bloody fluid in the abdomen
Laboratory examination: decreased red blood cell count, decreased hematocrit, decreased hemoglobin concentration
Auxiliary examination: ultrasound, CT, endoscopy, angiography

**Table 5:** Diagnosis of postoperative intra-abdominal infection.

Diagnostic basis
Clinical manifestations: high fever, chills, abdominal distension and other manifestations occurred 3 days after surgery and lasted for more than 24 hours
Laboratory examination: increased white blood cell count, may be accompanied by anemia, low protein
Imaging findings: presence of ascites
Diagnosis: abdominal puncture with purulent fluid or bacterial culture is positive

**Table 6:** Comparison of the two groups of patients.

Variables	Group A (*n* = 38)	Group B (*n* = 30)	*t*	*χ* ^2^	*p*
Age	61.88 ± 7.94	62.06 ± 8.93	-0.735		0.452
BMI (kg/m2)	23.92 ± 2.51	23.44 ± 2.45	0.718		0.435
Albumin (g/L)	34.89 ± 2.54	34.71 ± 4.08	0.319		0.754
Total bilirubin	116.89 ± 59.97	97.64 ± 30.11	1.921		0.062
Alanine aminotransferase	41.02 ± 17.35	42.54 ± 15.87	-0.325		0.735
Gender (male/female)	20/21	17/13		0.419	0.513
Diabetes (yes/no)	3/35	4/25		0.001	0.986
Chronic pancreatitis (yes/no)	2/36	5/23		0.062	0.802
Pancreatic texture (hard/soft)	7/31	6/21		0.002	0.954
Pancreatic duct diameter (thin/thick)	9/29	2/27		0.046	0.823
History of upper abdominal surgery (yes/no)	2/35	3/25		0.001	1.002
Intraoperative hemorrhage < 400mI (yes/no)	31/7	28/2		3.056	0.078
Pathological type (pancreatic/nonpancreatic)	3/35	7/24		2.203	0.132

**Table 7:** Comparison of intraoperative conditions between the two groups of patients.

Group	Group A	Group B	*t*	*p*
Operation time (h)	4.67 ± 0.91	5.64 ± 1.03	1.913	0.041
Number of lymph nodes removed	19.87 ± 2.32	17.85 ± 6.18	-1.432	0.235

**Table 8:** Comparison of postoperative complications between the two groups of patients.

Group	Group A (*n* = 38)	Group B (*n* = 30)	*χ* ^2^	*t*	*p*
Pancreas anastomosis time	32.13 ± 4.52	43.23 ± 4.31		-7.285	<0.001
Biochemical fistula	8	3	0.732		0.378
Grade B fistula	2	8	4.562		0.029
Biochemical fistula+grade B fistula	10	11	0.924		0.315
Biliary fistula	3	3	0.001		1.001
Postoperative hemorrhage	3	3	0.092		0.754
Abdominal infection	4	5	3.157		0.074
Delayed gastric emptying	5	2	0.565		0.441

## Data Availability

All data included in this study are available upon request by contact with the corresponding author.
